# Invasive vs. Non-Invasive Neuronal Signals for Brain-Machine Interfaces: Will One Prevail?

**DOI:** 10.3389/fnins.2016.00295

**Published:** 2016-06-27

**Authors:** Stephan Waldert

**Affiliations:** Sobell Department of Motor Neuroscience and Movement Disorders, University College London Institute of Neurology, University College LondonLondon, UK

**Keywords:** Brain computer interface (BCI), intracortical, action potentials, LFP, EEG, neuroprosthetics, microstimulation, Ethics

Brain-machine interfaces (BMI) translate neuronal activity of the brain into signals driving an external effector or affecting internal body parts and functions. Initially, their applications were seen in the field of rehabilitation and medical care for patients to restore social interaction or movement capabilities. Inspired by their success we can already witness the advent of bidirectional and commercial BMIs.

Contemporary BMIs allow for real-time control of prostheses (Gilja et al., 2015), communication (Chen et al., 2015) and “sensation” (O'doherty et al., 2011), notably, the cochlea implant could be considered as the most successful BMI. These applications exemplify that performance can be high but is far from natural interaction with the environment and success depends on manifold factors.

This Opinion is not about algorithms and paradigms but about possibilities and limitations of invasive vs. non-invasive means to *electrically* interface the brain, argued in the realm of BMIs for direct and intuitive motor control.

Current techniques allow to interface electric neuronal activity *in vivo* ranging from intracellular potentials over extracellular action potentials (APs) up to local field potentials (LFPs). These neurophysiological processes are inherently coupled: neurons can interact ephaptically and via electric synapses, spikes change LFPs via synaptic input which in turn influences spiking activity, electric fields of APs can influence LFPs directly without involvement of synaptic currents. Although the LFP is difficult to interpret (Einevoll et al., 2013), correlations between APs and LFPs vary (Buzsaki et al., 2012) and the information they convey can be independent (Belitski et al., 2008), this coupling may have given rise to discussions I have come across and which have triggered this Opinion: the misconception that, to a certain extent, information conveyed by invasive (APs/LFPs) vs. non-invasive (EEG) signals are similar enough for non-invasive signals, and thus non-invasive BMIs, not to be subject to intrinsic impediments.

Such speculations may have been nourished by studies showing similar performance for intracortical BMIs based on APs vs. LFPs (Mehring et al., 2003) as the latter are detectable by EEG techniques. Similar performance might be evident for multi-unit APs vs. high-frequency LFPs (>≈200 Hz), which contain extracellular fields of APs. However, also low/band-pass filtered LFPs below 8 Hz, generally free of such direct AP influences (Waldert et al., 2013), can show similar BMI performance as APs and are suitable for online BMIs (Stavisky et al., 2015). Importantly, this LFP component also carries information about movement parameters if recorded non-invasively (MEG, EEG; Waldert et al., 2008).

Non-invasive EEG yields lower performance than APs or LFPs (Waldert et al., 2009) but with the findings mentioned above and novel approaches: Could non-invasive BMIs catch up?

The source of neuronal signals extracted from EEG after thorough removal of noise, muscle, eye, and movement artifacts, are post-synaptic extracellular currents; in fact, the same currents that contribute to spike-free LFPs. Despite this common source, there are several differences, most of which well-documented, between invasive and non-invasive signals.

First, number and type of neurons: As electric fields produced by neurons decay exponentially with distance, the number of neurons that have to be simultaneously active in a confined area for the fields to superimpose and produce a detectable signal, is magnitudes smaller for LFP than EEG. Hence, the activity of small neuronal clusters is undetectable or recorded at a lower SNR with EEG. In addition, EEG signals are dominated by fields of pyramidal neurons as only their morphology (long, parallel dendrites) and high number in the cortex allow fields to add up and reach the scalp. In contrast, LFPs reflect a superposition of a variety of electrophysiological processes, those underlying EEG plus interneurons, APs, etc.

Second, signal composition: Tissue acts as a low-pass filter generally attenuating high-frequency signals to the extent that buries them in background noise. Hence, with the exception of AP bursts in neuronal populations (Waterstraat et al., 2015), non-invasive signals mainly allow analysis of low-frequency neuronal activity (<≈90 Hz, lower for dry EEG electrodes). Invasive signals convey information up to several kHz. Moreover, frequency-dependent phase shifts might be stronger when signals spread across larger distances (EEG) and might disintegrate temporal consistency across signal components.

Third, spatial distortion: The extracellular space is composed of media with different electrophysiological properties, which influence how fields spread before being detected as LFPs. On top of this, fields spread in the cerebrospinal fluid, skull, and scalp, causing further spatial distortion before reaching EEG electrodes. Sophisticated head models and algorithms in combination with high-density EEG montages mitigate distortion (Michel and Murray, 2012) for signals above a certain noise level. To be similar to invasive signals, such models might need to be obtained *in vivo* for each user individually, rely on stable sensor positions, necessitate finite-element analysis and run near real-time (BMI performance depends on small delays, Cunningham et al., 2011).

These limitations are intrinsic to EEG and cannot be practicably (or theoretically) overcome. However, EEG offers the paramount advantage to monitor large-scale neuronal activity of the entire brain adjacent to the neurocranium at a low cost and risk-free. Invasive recordings can be deeper but cannot cover the whole neocortex and are initially more laborious due to surgical interventions.

Invasive electrodes come in many forms: single electrodes, electrodes with multiple contacts at the tip or along the shaft, multi-electrode arrays (MEA), or combinations of these in different designs. Electrodes can have arbitrary lengths up to several cm or, for example, up to 1.5 mm (Utah, Blackrock Microsystems) or 10 mm (FMA, MicroProbes) in a MEA. Intracortical electrodes typically yield LFPs and detectable APs of 0–5 identifiable neurons per intact contact. Electrodes can be specifically targeted at arbitrary cerebral areas although accuracy decreases with implantation depth (unless aided by MRI and/or CT).

Nevertheless, for several reasons high implantation accuracy seems not to be crucial for invasive motor BMIs as long as contacts remain in gray matter. The general aim is to record APs and LFPs. In motor cortex, LFPs are recorded at different depths and convey information about movement parameters; recorded APs are faint at layer 1 and usually increase in amplitude with electrode depth up to layer 5 because the size of pyramidal cell somas tends to increase from layer 3 to 5, possibly to support the longer dendrites necessary to project to superficial (input) layers, and because layer 5 is the place of large corticospinal neurons, a main cortical output to control motor functions. This region may therefore often be targeted in invasive motor BMIs for high performance. Importantly, even MEAs with relatively short electrodes (Utah) should have access to this activity because: layer 4 is very thin in motor cortex (Rockel et al., 1980), floating MEAs sink into cortical tissue and APs of large pyramidal neurons can be recorded at several 100 μm distance (experimental and analytical experiences here in the Sobell Department, UCL). For deeper regions, like the anterior wall of the central sulcus, MEAs with longer electrodes can be used to follow layers into the sulcus.

Overall and in contrast to non-invasive signals, invasive signals reflect input to, local processing and output of cortical areas. They may even allow to deduce on intracellular states of neurons (Henze et al., 2000).

Hence, a main advantage of intracortical over non-invasive approaches are inherently possible higher information transfer rates. This and two further advantages are decisive for the future of motor BMIs: tuning and sensation.

BMI performance is still far from natural. After BMI initiation this is partly due to an undersampling of the neuronal network required for natural motor control. Performance then increases during BMI usage as the neurons' tuning “improves” (Carmena et al., 2003), i.e., plasticity enables the brain to learn to control the BMI (closed-loop). This works with arbitrary neurons (Fetz, 1969) and is facilitated by using already tuned neuronal activity (Ganguly and Carmena, 2009) accompanied by a transition from externally assisted to full brain control (Collinger et al., 2013). LFPs seem to be more stable (Flint et al., 2013; Perge et al., 2014), i.e., less easy to tune; probably as in contrast to spiking activity of some neurons, activity of a neuronal cluster needs to change coherently. Although possible (Okazaki et al., 2015), this holds even more so for EEG.

Feedback in closed-loop BMIs has been mainly visual or acoustic. Such inadequate feedback also accounts for low BMI performance as the absence of direct forms of feedback (touch and proprioception) impoverishes information contained in brain signals (Galan et al., 2014) and can disturb the generation of appropriate motor commands (Galan and Baker, 2015). Researchers have begun to employ intracortical microstimulation to establish a direct BMI input channel (Klaes et al., 2014) with possible long-term stability (Callier et al., 2015). This should eventually improve performance as feedback may be delivered specifically to task-relevant cortical areas, which closes the output-feedback loop adequately. As electrodes may be used for stimulation and recording; stimulation could be adapted to ongoing brain activity to improve efficacy.

In contrast to non-invasive BMIs, the great opportunity offered by invasive BMIs thus lies in accurate control, a prerequisite for user acceptance, combined with restoration of somatosensation: Prostheses will be controlled using high-dimensional BMI output signals (Wodlinger et al., 2015) while at the same time BMI input signals, obtained from skin prostheses (Kim et al., 2014) during interaction with the environment, will be transmitted to cortical sensory areas. Providing such information may remain far off evoking natural percepts but the brain will learn to make use of such artificial input channels.

User acceptance is lower for invasive than non-invasive BMIs (Blabe et al., 2015). Invasive BMIs will for many years remain to be used in patients, either for research or if no other remedy is available. Present commercial BMIs are all non-invasive.

This lower acceptance mainly arises from medical concerns related to neurosurgery and the implant. Such risks are clearly not negligible but seem to be partly overrated. For example, validation of DBS showed that complications are rare and, with appropriate procedures, are reduced to 0.9% transient and no permanent deficits (Zrinzo et al., 2012). Even if multiple subpial transection, a series of long cuts in gray matter used to treat epilepsy, is performed in the primary motor cortex, patients are left with no permanent motor deficits (Blount et al., 2004). Implanting electrode arrays for invasive motor BMIs should appear innocuous against this procedure. They have been used in many laboratories for years now and also here in the Sobell Department, UCL, we have not experienced any motor deficits after array implantations. Medical concerns might subside with better awareness of such evidence.

It is now crucial to overcome current challenges of invasive BMIs: better understanding of the “neuronal code,” implant miniaturization, wireless signal transmission (Borton et al., 2013), implants charged from outside (Ho et al., 2014) or by harvesting energy from the body (Hannan et al., 2014). BMIs need to be asynchronous for unrestricted control, adaptable to unstable signals and require better sensory-motor prostheses. A major challenge of intracortical implants is biocompatibility, time-dependent degradation of recording quality, and eventually implant failure due to tissue damage during implantation, array micromotion, and a breach of the blood-brain-barrier triggering glial scarring, neurodegeneration, and neuronal death. Only few APs are still recorded years after implantation (Hochberg et al., 2012). LFPs also deteriorate but might show better long-term stability (Flint et al., 2013; Perge et al., 2014). To increase longevity and yield, electrodes need to be reduced in size, coated with neurointegrative, anti-inflammatory factors (Gunasekera et al., 2015), and/or redesigned (e.g., carbon nanotubes, Vitale et al., 2015; Lopez et al., 2016).

As an invasive but extracortical technique, miniaturized ECoG causes lesser cortical tissue damage/irritation and allows for epicortical recordings of LFPs at high spatial resolution and, as recently shown, also of spiking activity (Khodagholy et al., 2015). Benefits derivable from such advances, especially regarding increased information transfer rates, biocompatibility, and long-term signal stability (Chao et al., 2010) over years, are being investigated and might be decisive for the development of future BMIs.

Once invasive BMIs are fully body-embeddable and their benefits outweigh concerns, they might become acceptable to the majority (of patients). However, other, non-medical concerns have to be addressed as well. As invasive BMIs allow access to the brain, i.e., the individual as such, it is necessary to discuss (and regulate) socio-ethical issues: privacy, “mind reading,” remote control, brain enhancement, which accuracy legitimates control of potentially hazardous devices, liability, and eventually self-perception and perception through others.

This Opinion is not a polemic against EEG. EEG is a prime tool for many applications, e.g., medical, rehabilitation, current BMIs for communication.

The conclusion of this Opinion is that once technical, socio-ethical, and neuroscientific challenges are resolved, user concerns might subside, and invasive BMIs (using primarily intracortical and potentially epicortical recordings) will prevail in most applications; certainly those for restoration of motor functions and perhaps even in applications not medically indicated.

## Author contributions

SW conceived and wrote the paper.

## Funding

This work was supported by the Wellcome Trust (WT102849MA).

### Conflict of interest statement

The author declares that the research was conducted in the absence of any commercial or financial relationships that could be construed as a potential conflict of interest.
